# Association between handgrip strength and heart failure in adults aged 45 years and older from NHANES 2011–2014

**DOI:** 10.1038/s41598-023-31578-9

**Published:** 2023-03-20

**Authors:** Run-Min Li, Guo-Hua Dai, Hui Guan, Wu-Lin Gao, Li-Li Ren, Xing-Meng Wang, Hui-Wen Qu

**Affiliations:** 1 First Clinical Medical College, Shandong University of Traditional Chinese Medicine, Jinan, China; 2 Department of Geriatrics, Affiliated Hospital of Shandong University of Traditional Chinese Medicine, 16369 Jing-Shi Road, Jinan, 250014 China; 3 College of Traditional Chinese Medicine, Shandong University of Traditional Chinese Medicine, Jinan, China

**Keywords:** Epidemiology, Risk factors, Population screening, Heart failure

## Abstract

Growing evidence indicates that handgrip strength (HGS) is a conspicuous marker for assessing some diseases affecting middle-aged and elderly individuals. However, research regarding HGS and heart failure (HF) is sparse and controversial. Hence, we aimed to investigate the association between HGS and HF among adults aged 45 years and older in the United States. In this cross-sectional study, we included 4524 adults older than 45 years who were part of the National Health and Nutrition Examination Survey. A generalized additive model was used to estimate the association between HGS and HF. Age, gender, race, income, education, body mass index, smoking status, drinking status, diabetes, hypertension, stroke, vigorous physical activity, total energy intake, total protein intake, total sugars intake, and total fat intake covariates were adjusted using multiple regression models. And further subgroup analysis was conducted. We documented 189 cases of HF, including 106 men and 83 women. HGS was negatively associated with HF after adjusting for all the covariates (odds ratio = 0.97, 95% confidence interval = 0.96–0.99; *P* < 0.001). Compared with the lowest quintile, the highest quintile was associated with an 82% lower incidence of HF (odds ratio = 0.18, 95% confidence interval = 0.08–0.43; *P* < 0.001). Subgroup analysis showed that the results remained stable. In US adults older than 45, HGS was negatively associated with HF after adjusting for covariates. This finding had the potential to draw attention to the physiological and pathological effects of decreased muscle function on HF and may influence further prospective studies with intervention trials.

## Introduction

Along with the global social structure of population aging, the trend of aging brings about an increase in the overall incidence and prevalence of heart failure (HF). Currently, the incidence of HF in Europe is about 3/1000 person-years (all age groups) or about 5/1000 person-years in adults, the increase in overall incidence is mainly associated with people over 85, with a limited contribution from people under 55^1,2^. At the same time, data from the Heart Failure Association (HFA) of the European Society of Cardiology (ESC) showed an overall HF prevalence of 17 cases per 1000 people, the prevalence is approximately 1% for those aged < 55 years and > 10% for those aged 70 years or older^1,3^. In the United States, HF currently affects 6 million people, and the direct costs of HF treatment are expected to cost up to $30.7 billion and will double by 2030^4,5^. Importantly, studies have shown that even patients with mild symptoms may still have a high risk of hospitalization and death^6^. A study also showed that the most clinically stable HF patients, who had never had a prior or remote HF hospitalization, still had high absolute rates of cardiovascular death and hospitalization during the course of trial^7^. There is a need to explore more prevention strategies for HF, which necessitates a better understanding of the association between risk factors and HF.

Handgrip strength (HGS) is a quick and straightforward measure of muscle function and is closely related to overall muscle strength^8^. Age-related reductions in overall muscle strength are associated with all-cause mortality and other adverse clinical events in middle-aged or elderly people and can be characterized by HGS^9–11^. For example, studies have shown that reduced HGS increases the risk of all-cause mortality and cancer^12^. Overall, HGS is associated with cardiovascular mortality and the incidence of cardiovascular disease^12–14^, but data on the association between HGS and HF remains sparse and controversial. For instance, Segar et al. suggested that decreased HGS had a nonsignificant association with a higher incident risk of both reduced and preserved ejection fraction heart failure^15^. At the same time, Hauptman et al. found that changes in HGS were not associated with 30-day HF readmission^16^. In contrast, a cohort study showed that relative HGS (absolute values of HGS divided by weight in kilograms) was inversely associated with the risk of heart failure^17^. A case–control study suggested that HGS is an ideal indicator of risk stratification in patients with HF^18^. In a meta-analysis, HGS was considered to be an independent predictor of admissions for HF^19^.

Therefore, in this study, we examined the associations of HGS with HF among US adults aged 45 years and older using samples from a database of a multiracial population and tried to explain the mechanism.

## Methods

### Ethics statement

The use of the dataset from the National Health and Nutrition Examination Survey (NHANES) was approved by the National Center for Health Statistics (NCHS) Institutional Review Board in compliance with the revised Declaration of Helsinki. Informed consent was obtained from all participants before data collection. All the methods were carried out in accordance with the relevant guidelines and regulations of the NCHS Institutional Review Board.

### Data sources

The NHANES is a nationally representative survey conducted by the NCHS. It adopted a stratified, multistage probability cluster sampling design to select representative samples from United States civilians and assess their health or nutritional status. The survey data and methodological details about the NHANES are available at www.cdc.gov/nchs/nhanes/.

### Study design and participants

This study followed the Strengthening the Reporting of Observational Studies in Epidemiology (STROBE) reporting guideline. As a retrospective cross-sectional study, no direct contact was performed with the participants, so the privacy risk was minimal. The deidentified data were extracted from the 2011–2014 NHANES cycles since the information on HGS measurement was only provided in these cycles. In order to strictly screen the included participants, the following exclusion criteria were used: participants with cognitive impairment and depression were excluded because these conditions may cause abnormal HGS data^20,21^; participants missing data for HF, HGS, or other covariates; the age of participants under 45 years old. Data were analyzed from May to July 2022.

### HGS measurement and diagnosis of HF

The exposure variable was HGS, which was measured using a handgrip dynamometer (Model T.K.K.5401). The HGS measurement protocol was explained and demonstrated to the participant by a qualified examiner. The examiner next adjusted the handgrip size of the dynamometer to the participant’s hand size and requested the participant to try squeezing the dynamometer for a practice test. The purpose of the practice test was to check whether the participant understood the procedure and whether the handgrip size was properly adjusted. After practice, the participant was instructed to squeeze the dynamometer as hard as possible with one hand while exhaling to prevent intrathoracic pressure buildup. The test was then repeated for the other hand. Each hand was evaluated three times, with a 60-s interval of rest between measurements on the same hand. The HGS was calculated as the sum of the largest reading from each hand and expressed in kilograms. This variable was not calculated for participants who only performed the test on one hand, such as participants with “missing arm, hand, or thumb,” “hand paralysis,” “wearing a cast on wrist or hand” or “other (specify)”. Unless the participant was physically disabled, the handgrip test was conducted in a standing posture. If the paraplegic participant is not able to obtain the proper testing form in the assigned sitting position, they will be excluded from the handgrip test. Detailed descriptions of the HGS measurement protocol are provided in the NHANES “Muscle Strength Procedures Manual”^22^.

The outcome variable was HF. In the NHANES, HF data were provided by a self-reported personal interview. Participants were considered to have HF if they answered yes to the question “Has a doctor or other health professional ever told you that you had heart failure^23^? Although the method of defining the main outcome with the results of the questionnaire survey had certain ambiguity. However, given the lack of B-type natriuretic peptide (BNP), N-terminal pro-B-type natriuretic peptide (NT-proBNP), cardiac troponin, electrocardiogram, or cardiac imaging in the NHANES database, it was difficult to make a definite diagnosis of heart failure. Some existing literature also supported the use of questionnaires as a diagnostic method for heart failure in NHANES participants^24–26^.

### Covariates

Variables thought to be confounders based on existing literature and clinical judgment were included^4,20,23,27,28^. In this study, covariates included demographic data (age, gender, race, education, income, body mass index), a questionnaire on medical history (diabetes mellitus, hypertension, stroke), lifestyle characteristics (smoking status, drinking status, vigorous physical activity), and nutrient intake situation (total energy intake, total protein intake, total sugars intake, and total fat intake).

In NHANES, information on self-reported race and ethnicity was derived from responses to survey questions on race. Educational was divided into 3 levels (high school or less, some college, and college graduate or higher). BMI is calculated from a given height and weight of participants. As used by US government departments to report NHANES dietary and health data, we categorized family income into the following 3 levels based on the family poverty income ratio: low income (≤ 1.3), medium income (> 1.3 to 3.5), and high income (> 3.5)^29^. Diabetes mellitus, hypertension, stroke, and vigorous physical activity were defined based on the self-reported questionnaire. Smoking status was categorized into the following 3 groups: never smoked (or smoked < 100 cigarettes), former smoker (smoked at least 100 cigarettes but has quit), and current smoker. Drinking status was determined by the survey question, “In any 1 year, have you had at least 12 drinks of any type of alcoholic beverage?” Participants who answered “yes” were defined as alcohol drinkers^30^. The vigorous physical activity was determined by a questionnaire, “Do you do any vigorous-intensity sports, fitness, or recreational activities that cause large increases in breathing or heart rate like running or basketball for at least 10 min continuously?” The option was “Yes” or “No”. Estimates of dietary intake data were assessed from the 24-h dietary recall, which utilized the Automated Multiple Pass Method performed by trained interviewers in the Mobile Examination Center^31^. This multi-step procedure provided for enhanced accuracy of the intakes of foods and beverages reported as ingested from midnight to midnight of the prior day^32,33^. Nutrient intakes were estimated from the Food and Nutrient Database for Dietary Studies as well as the Food Patterns Equivalents Database, respectively, to estimate intakes on the day of record^34^. The total energy intake was measured in kilocalories (kcal), total protein intake, total sugars intake, and total fat intake were measured in grams (gm). The data acquisition process for all the covariates can be found at www.cdc.gov/nchs/nhanes/.

### Statistical analysis

Descriptive analysis was applied to all participants’ data. Categorical variables are expressed as proportions (%). Continuous variables are expressed as the mean and standard deviation (SD) or median and interquartile range (IQR), as appropriate. Student’s t-test and the chi-square test were used for continuous variables and categorical variables, respectively, to assess differences in clinical characteristics.

**Figure 1:**
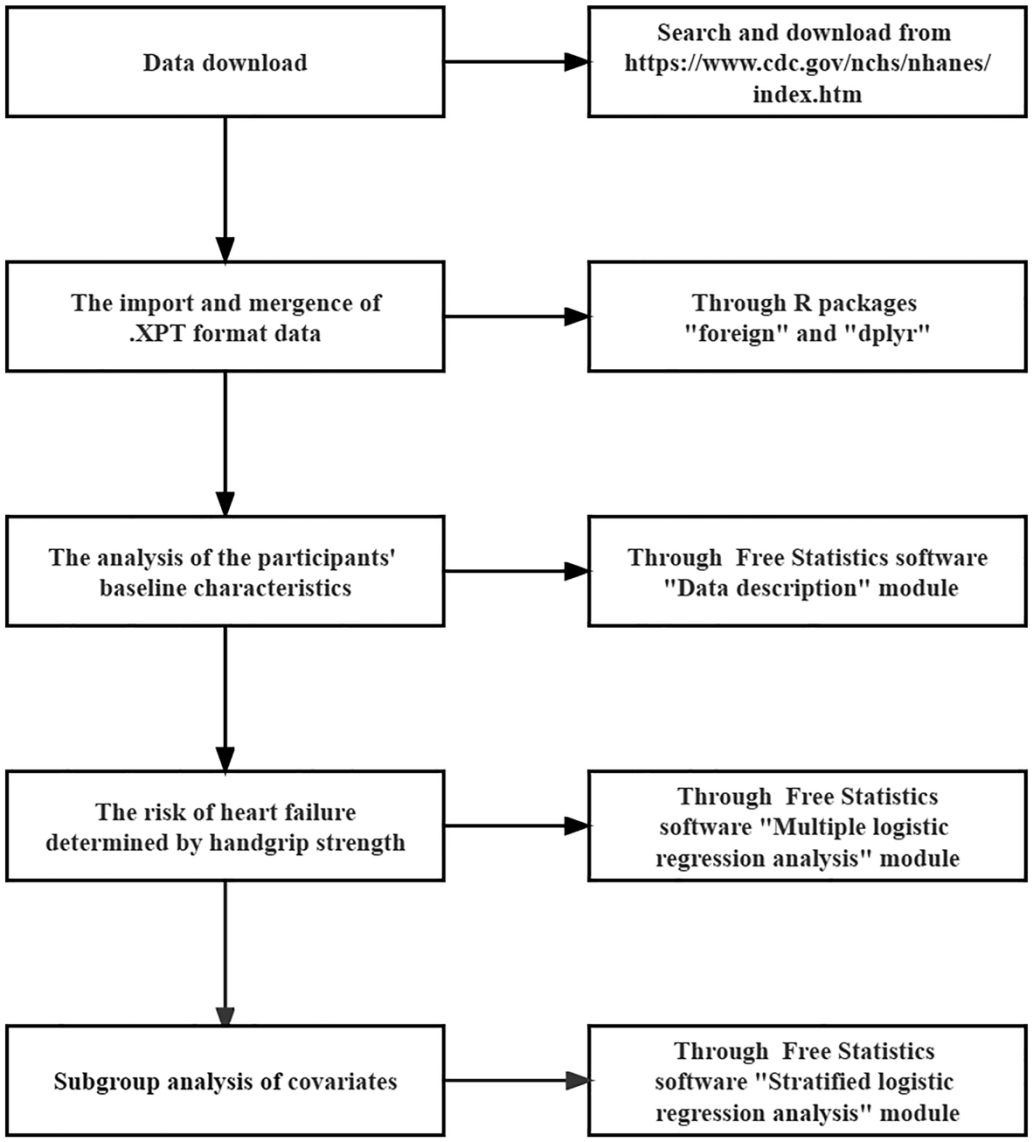
The block diagram of the main data analysis process.

Odds ratios (ORs) and 95% confidence intervals (CIs) were calculated for HGS with HF using multiple logistic regression models. Age, gender, race, income, education, BMI, smoking status, drinking status, hypertension, diabetes, stroke, vigorous physical activity, total energy intake, total protein intake, total sugars intake, and total fat intake covariates were adjusted. A generalized additive model was used to study the association between HGS and HF. Age levels were classified into ≥ 60 years old and < 60 years old, according to the World Health Organization’s definitions of middle age and old age. BMI levels were divided into two groups by 25. Each nutrient intake was divided into three equal subgroups. Subgroup analysis of age levels, gender, race, income levels, education levels, BMI levels, smoking status, drinking status, hypertension, diabetes, stroke, vigorous physical activity, total energy intake levels, total protein intake levels, total sugars intake levels, and total fat intake levels covariates were performed using stratified logistic regression models. Interaction across subgroups was tested using the likelihood ratio test. A *P* < 0.05 was considered statistically significant. Relevant methodological descriptions could be found in the literature^30,35^. The import and mergence of original data were performed with the statistical software R packages (“foreign” and “dplyr”) (http://www.R-project.org, The R Foundation). The Free Statistics software version 1.4 (Freeclinical Medical Technology Co, Ltd. http://www.clinicalscientists.cn/freestatistics/) was utilized for descriptive analysis, multiple logistic regression analysis, and subgroup analysis. By setting the function menu and calling the built-in R language packages, the software performed data analyses. The initialization file (settings file) was where the software stored the outcomes of repeated data processing. Figure 1 depicted the main data analysis process.

## Results

### Baseline characteristics of the study participants by categories of HGS

**Figure 2:**
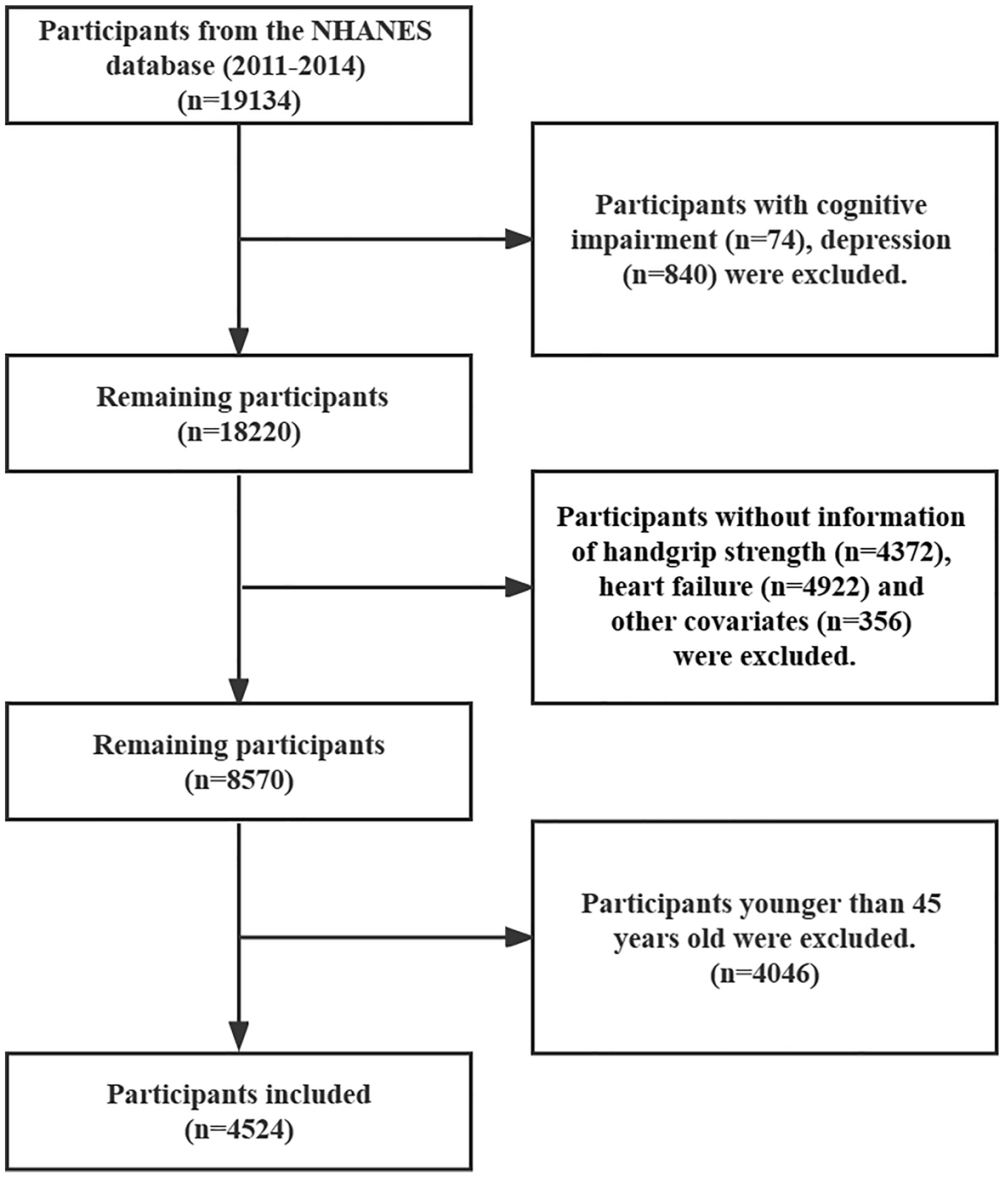
Flow chart of sample selection from the NHANES 2011–2014.

The flowchart for participant enrollment is presented in Fig. 2. Participants with cognitive impairment (n = 74) and depression (n = 840) were excluded. A total of 4524 participants aged 45 years and older remained after the exclusion of 4372 subjects with missing handgrip strength data, 4922 subjects with missing HF data, and 356 subjects with missing other covariates.

Among the 4524 participants from the study, 189 participants were diagnosed with HF. The baseline characteristics of all participants are shown in Table 1. The average age of the study participants was 61.78 years. Males accounted for 50.22% of the total study population. All the variables were significantly different among persons classified into the different quintiles of HGS. The lowest quintile of HGS was ≤ 48.40 kg; the 2nd quintile was ≥ 48.50 and < 58.10 kg; the 3rd quintile was ≥ 58.20 and < 70.20 kg; the 4th quintile was ≥ 70.30 and < 85.70 kg; and the highest quintile was ≥ 85.80 kg. Compared with participants in the HGS quintile 1 group, the other quintile groups were younger, more likely to be male, had higher income, education, BMI, and nutrient intake, and had lower rates of diabetes, hypertension, and stroke. However, smoking, drinking, and vigorous physical activity rates were higher.

**Table 1:** Description of 4524 participants included in the present study.

Variable	All participants	Handgrip strength (kg)					*P* value
		Q1 (≤ 48.40)	Q2 (48.50–58.10)	Q3 (58.20–70.20)	Q4 (70.30–85.70)	Q5 (≥ 85.80)	
Participants (n)	4524	902	895	917	905	905	
Heart failure n(%)							< 0.01
No	4335 (95.82)	845 (93.68)	851 (95.08)	874 (95.31)	870 (96.13)	895 (98.90)	
Yes	189 (4.18)	57 (6.32)	44 (4.92)	43 (4.69)	35 (3.87)	10 (1.10)	
Age (years)	61.78 ± 10.67	68.74 ± 10.13	62.28 ± 10.25	60.47 ± 10.53	60.91 ± 10.21	56.53 ± 8.25	< 0.01
Gender n(%)							< 0.01
Female	2252 (49.78)	826 (91.57)	734 (82.01)	545 (59.43)	140 (15.47)	7 (0.77)	
Male	2272 (50.22)	76 (8.43)	161 (17.99)	372 (40.57)	765 (84.53)	898 (99.23)	
Race n(%)							< 0.01
Mexican American	428 (9.46)	90 (9.98)	92 (10.28)	78 (8.50)	82 (9.06)	86 (9.50)	
Non-Hispanic White	2037 (45.03)	462 (51.22)	406 (45.36)	392 (42.75)	367 (40.55)	410 (45.30)	
Non-Hispanic Black	1127 (24.91)	134 (14.85)	193 (21.57)	258 (28.14)	248 (27.40)	294 (32.49)	
Other	932 (20.60)	216 (23.95)	204 (22.79)	189 (20.61)	208 (22.99)	115 (12.71)	
BMI (kg/m^2^)	29.21 ± 6.48	28.58 ± 6.84	29.07 ± 6.69	29.62 ± 6.95	29.03 ± 6.08	29.75 ± 5.69	< 0.01
Income n(%)							< 0.01
PIR ≤ 1.3	1342 (29.67)	327 (36.25)	265 (29.61)	258 (28.13)	270 (29.84)	222 (24.53)	
1.3 < PIR ≤ 3.5	1589 (35.12)	344 (38.14)	310 (34.64)	331 (36.10)	308 (34.03)	296 (32.71)	
PIR > 3.5	1593 (35.21)	231 (25.61)	320 (35.75)	328 (35.77)	327 (36.13)	387 (42.76)	
Education n(%)							< 0.01
High school or less	1005 (22.21)	263 (29.16)	168 (18.77)	194 (21.16)	208 (22.98)	172 (19.01)	
Some college	2323 (51.35)	471 (52.22)	475 (53.07)	480 (52.34)	432 (47.74)	465 (51.38)	
College graduate or higher	1196 (26.44)	168 (18.62)	252 (28.16)	243 (26.50)	265 (29.28)	268 (29.61)	
Diabetes n(%)							< 0.01
No	3705 (81.90)	701 (77.72)	733 (81.90)	768 (83.75)	734 (81.10)	769 (84.97)	
Yes	819 (18.10)	201 (22.28)	162 (18.10)	149 (16.25)	171 (18.90)	136 (15.03)	
Hypertension n(%)							< 0.01
No	2739 (60.54)	464 (51.44)	585 (65.36)	577 (62.92)	555 (61.33)	558 (61.66)	
Yes	1785 (39.46)	438 (48.56)	310 (34.64)	340 (37.08)	350 (38.67)	347 (38.34)	
Stroke n(%)							< 0.01
No	4304 (95.14)	813 (90.13)	862 (96.31)	878 (95.75)	869 (96.02)	882 (97.46)	
Yes	220 (4.86)	89 (9.87)	33 (3.69)	39 (4.25)	36 (3.98)	23 (2.54)	
Smoking status n(%)							< 0.01
Never smoker	2360 (52.17)	560 (62.08)	528 (58.99)	485 (52.89)	377 (41.66)	410 (45.31)	
Former smoker	1420 (31.38)	253 (28.05)	249 (27.83)	270 (29.44)	358 (39.56)	290 (32.04)	
Current smoker	744 (16.45)	89 (9.87)	118 (13.18)	162 (17.67)	170 (18.78)	205 (22.65)	
Drinking status n(%)							< 0.01
Never drinker	1243 (27.48)	411 (45.57)	304 (33.97)	264 (28.79)	167 (18.45)	97 (10.72)	
Former drinker	3103 (68.59)	453 (50.22)	556 (62.12)	609 (66.41)	708 (78.24)	777 (85.85)	
Current drinker	178 (3.93)	38 (4.21)	35 (3.91)	44 (4.80)	30 (3.31)	31 (3.43)	
Vigorous physical activity n(%)							< 0.01
No	3868 (85.50)	864 (95.79)	827 (92.40)	802 (87.46)	745 (82.32)	630 (69.61)	
Yes	656 (14.50)	38 (4.21)	68 (7.60)	115 (12.54)	160 (17.68)	275 (30.39)	
Total energy intake (kcal)	1988.52 ± 893.59	1644.10 ± 745.07	1742.65 ± 693.67	1936.20 ± 767.73	2196.17 ± 955.57	2420.31 ± 1022.18	< 0.01
Total protein intake (gm)	78.31 ± 39.57	63.88 ± 31.98	68.20 ± 30.42	75.85 ± 36.33	87.79 ± 42.73	95.71 ± 45.03	< 0.01
Total sugars intake (gm)	102.37 ± 67.07	90.09 ± 58.87	93.16 ± 57.60	98.91 ± 62.93	108.76 ± 69.47	120.86 ± 79.41	< 0.01
Total fat intake (gm)	76.47 ± 43.54	62.79 ± 37.31	67.09 ± 36.18	74.64 ± 38.68	84.37 ± 47.68	93.36 ± 48.87	< 0.01

Data presented are mean ± SD or n(%).

*BMI* body mass index, *PIR* poverty income ratio.

The *P* value < 0.05 represents the significance of the comparison among groups.

### Association between HGS and HF

**Figure 3:**
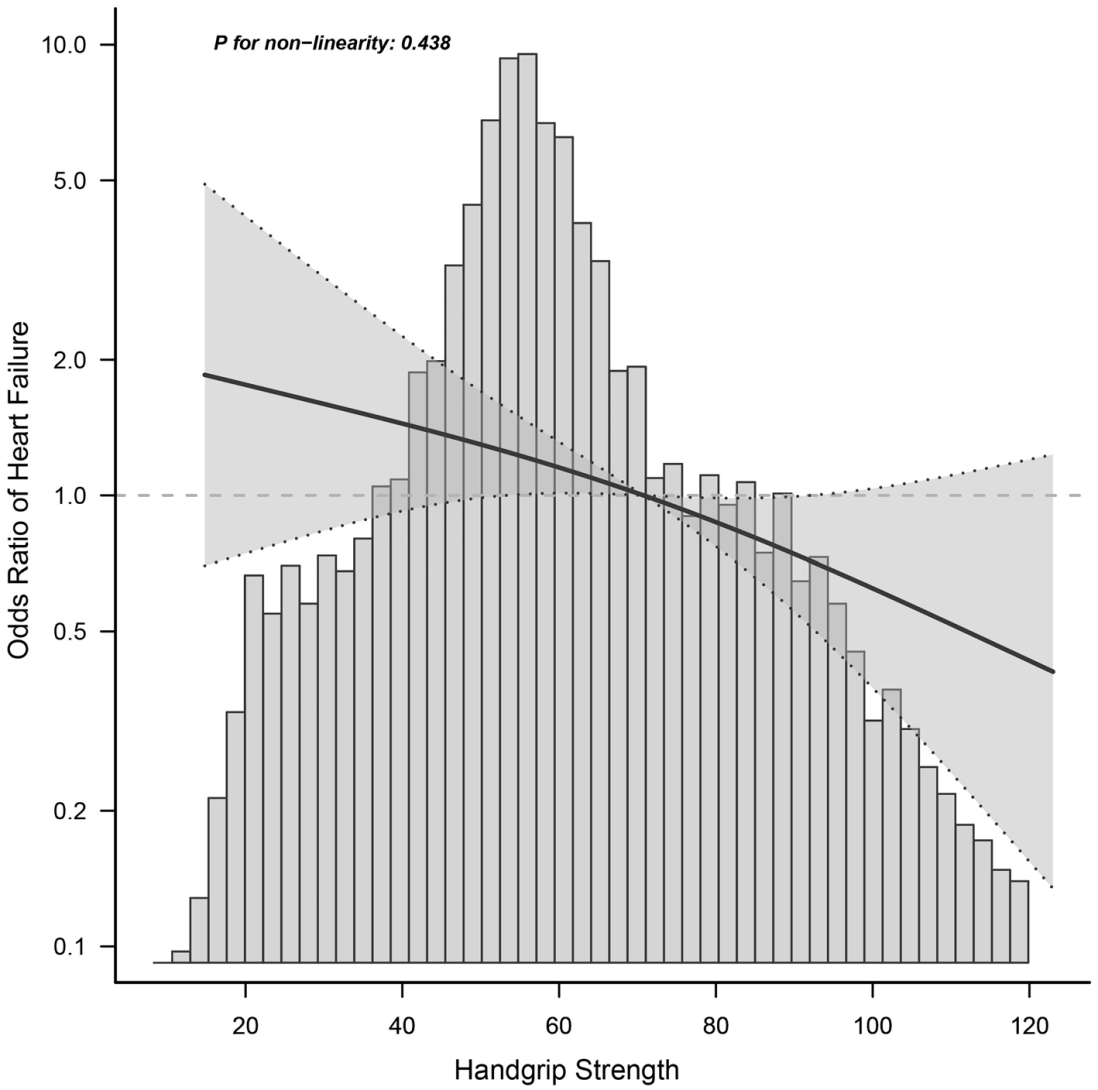
The non-linearity test of handgrip strength and heart failure.

The generalized additive model was utilized to test the non-linearity of HGS and HF. As shown in Fig. 3, there was a linear association between HGS and HF (*P* for non-linearity = 0.438 > 0.05).

Table 2 shows the ORs and 95% CIs for the risk of HF determined by HGS. The unadjusted model omits any adjustment for covariables. The adjusted model I adjusts for age, race, gender, income, and education. The adjusted model II adjusts for age, race, gender, income, education, BMI, smoking status, drinking status, diabetes, hypertension, stroke, vigorous physical activity, total energy intake, total protein intake, total sugars intake, and total fat intake. When analyzed in continuous form, HGS was significantly associated with the incidence of HF. This association was found in the unadjusted model (OR = 0.98, 95% CI = 0.97–0.98), adjusted model I (OR = 0.97, 95% CI = 0.96–0.98), and adjusted model II (OR = 0.97, 95% CI = 0.96–0.99).

**Table 2:** Association of HGS with HF.

	Unadjusted model	Adjusted model I	Adjusted model II
HGS (kg)	0.98 (0.97–0.98)	0.97 (0.96–0.98)	0.97 (0.96–0.99)
HGS quintiles			
Q1 (≤ 48.40)	1(Ref)	1(Ref)	1(Ref)
Q2 (48.50–58.10)	0.77 (0.51–1.15)	0.99 (0.64–1.54)	1.12 (0.70–1.77)
Q3 (58.20–70.20)	0.73 (0.49–1.10)	0.75 (0.46–1.23)	0.89 (0.53–1.49)
Q4 (70.30–85.70)	0.60 (0.39–0.92)	0.47 (0.27–0.84)	0.53 (0.29–0.98)
Q5 (≥ 85.80)	0.17 (0.08–0.33)	0.16 (0.07–0.36)	0.18 (0.08–0.43)
*P* for trend	< 0.001	< 0.001	< 0.001

Data presented are ORs and 95% CIs. The adjusted model I adjusts for age, race, gender, income, and education. Adjusted model II adjusts for adjust I + BMI, smoking status, drinking status, diabetes, hypertension, stroke, vigorous physical activity, total energy intake, total protein intake, total sugars intake, and total fat intake. “*P* for trend” is mainly used to test whether there is a certain linear change trend between the change in the exposure variable of HGS and the change in the outcome variable of HF.

When treated as a categorical variable, in the unadjusted model, there was a decreasing risk of developing HF as the quintile of HGS increased (*P* for trend < 0.001). Compared with those in the lowest quintile, participants who had a measurement of HGS in the highest quintile had an 83% decreased risk of the development of HF (OR = 0.17, 95% CI = 0.08–0.33). After adjustment for age, race, gender, income, education, BMI, smoking status, drinking status, diabetes, hypertension, stroke, vigorous physical activity, total energy intake, total protein intake, total sugars intake, and total fat intake, the odds ratios were (OR = 1.12, 95% CI = 0.70–1.77), (OR = 0.89, 95% CI = 0.53–1.49), (OR = 0.53, 95% CI = 0.29–0.98), and (OR = 0.18, 95% CI = 0.08–0.43) for HGS quintiles 2–5, respectively (*P* for trend < 0.001).

### Subgroup analyses of the association between HGS and HF

To determine whether the association between HGS and HF was stable in different subgroups, we performed stratified analyses and interactive analyses (Table 3). No interactive role was found in the association between HGS and HF (*P* for interaction > 0.05).

**Table 3:** Subgroup analyses of the association between HGS and heart failure.

Confounding factor	HGS quintiles					*P* for trend	*P* for interaction
Category	Q1	Q2	Q3	Q4	Q5		
	(≤ 48.40)	(48.50–58.10)	(58.20–70.20)	(70.30–85.70)	(≥ 85.80)		
Age levels (years)							0.18
n ≤ 60	1(Ref)	0.71 (0.21–2.40)	0.35 (0.09–1.30)	0.18 (0.04–0.83)	0.01 (0–0.12)	< 0.01	
n > 60	1(Ref)	1.15 (0.69–1.91)	1.04 (0.58–1.86)	0.64 (0.32–1.26)	0.36 (0.14–0.91)	0.03	
Gender n(%)							0.51
Female	1(Ref)	0.88 (0.49–1.59)	0.64 (0.28–1.44)	0.27 (0.03–2.15)	0 (0-Inf)	0.15	
Male	1(Ref)	2.72 (0.95–7.83)	2.02 (0.73–5.60)	1.08 (0.38–3.03)	0.34 (0.10–1.14)	< 0.01	
Race n(%)							0.20
Mexican American	1(Ref)	1.03 (0.15–6.84)	1.38 (0.17–11.02)	0.21 (0.02–2.63)	0 (0–Inf)	0.04	
Non-Hispanic White	1(Ref)	0.93 (0.49–1.77)	0.98 (0.48–2.00)	0.43 (0.18–1.03)	0.11 (0.03–0.46)	< 0.01	
Non-Hispanic Black	1(Ref)	0.67 (0.25–1.83)	0.51 (0.18–1.43)	0.68 (0.22–2.12)	0.39 (0.10–1.55)	0.18	
Other	1(Ref)	4.02 (0.65–24.81)	0.24 (0.01–4.42)	1.69 (0.14–19.91)	0 (0-Inf)	0.20	
Income levels n(%)							0.20
PIR ≤ 1.3	1(Ref)	0.99 (0.43–2.32)	1.75 (0.73–4.21)	1.36 (0.46–4.00)	0.80 (0.20–3.24)	0.82	
1.3 < PIR ≤ 3.5	1(Ref)	1.15 (0.57–2.32)	0.62 (0.27–1.43)	0.27 (0.09–0.76)	0.11 (0.02–0.50)	< 0.01	
PIR > 3.5	1(Ref)	1.22 (0.40–3.69)	0.55 (0.16–1.87)	0.37 (0.10–1.35)	0.05 (0.01–0.38)	< 0.01	
Education levels n(%)							0.74
High school or less	1(Ref)	1.08 (0.46–2.55)	0.87 (0.31–2.43)	0.36 (0.10–1.31)	0.46 (0.10–2.09)	0.15	
Some college	1(Ref)	1.15 (0.61–2.15)	0.93 (0.46–1.87)	0.68 (0.30–1.56)	0.11 (0.03–0.40)	< 0.01	
College graduate or higher	1(Ref)	0.56 (0.14–2.15)	0.44 (0.11–1.81)	0.20 (0.04–1.01)	0.09 (0.01–0.78)	0.02	
BMI levels (kg/m^2^)							0.73
n ≤ 25	1(Ref)	0.75 (0.28–2.04)	0.34 (0.10–1.20)	0.32 (0.08–1.26)	0.21 (0.04–1.22)	0.04	
n > 25	1(Ref)	1.29 (0.75–2.20)	1.15 (0.64–2.09)	0.59 (0.29–1.20)	0.18 (0.06–0.49)	< 0.01	
Diabetes n(%)							0.96
No	1(Ref)	1.30 (0.71–2.37)	1.10 (0.56–2.16)	0.60 (0.27–1.34)	0.25 (0.08–0.73)	0.01	
Yes	1(Ref)	0.78 (0.37–1.65)	0.64 (0.27–1.53)	0.43 (0.16–1.16)	0.11 (0.02–0.50)	< 0.01	
Hypertension n(%)							0.52
No	1(Ref)	1.35 (0.70–2.61)	0.75 (0.35–1.64)	0.53 (0.22–1.30)	0.12 (0.03–0.52)	< 0.01	
Yes	1(Ref)	0.90 (0.46–1.75)	0.98 (0.48–1.99)	0.48 (0.21–1.14)	0.21 (0.07–0.63)	< 0.01	
Stroke n(%)							0.79
No	1(Ref)	1.12 (0.68–1.84)	0.77 (0.43–1.37)	0.53 (0.28–1.02)	0.17 (0.07–0.42)	< 0.01	
Yes	1(Ref)	1.11 (0.26–4.69)	2.00 (0.45–8.82)	0.52 (0.06–4.23)	0.18 (0.01–3.46)	0.40	
Smoking status n(%)							0.54
Never smoker	1(Ref)	0.71 (0.37–1.37)	0.41 (0.18–0.91)	0.20 (0.07–0.53)	0.07 (0.02–0.27)	< 0.01	
Former smoker	1(Ref)	1.51 (0.66–3.50)	1.98 (0.84–4.66)	1.22 (0.46–3.27)	0.33 (0.07–1.53)	0.38	
Current smoker	1(Ref)	2.58 (0.59–11.23)	1.09 (0.21–5.54)	0.76 (0.13–4.64)	0.31 (0.04–2.57)	0.13	
Drinking status n(%)							0.95
Never drinker	1(Ref)	0.95 (0.46–1.99)	1.02 (0.42–2.47)	0.21 (0.04–1.10)	0.55 (0.11–2.74)	0.23	
Former drinker	1(Ref)	1.19 (0.63–2.25)	0.90 (0.45–1.80)	0.62 (0.29–1.33)	0.15 (0.05–0.43)	< 0.01	
Current drinker	1(Ref)	1.69 (0.26–10.75)	0.86 (0.11–6.40)	0.62 (0.05–7.19)	0 (0-Inf)	0.19	
Vigorous physical activity n(%)							0.57
No	1(Ref)	1.07 (0.66–1.73)	0.84 (0.48–1.45)	0.59 (0.31–1.11)	0.17 (0.06–0.46)	0.01	
Yes	1(Ref)	2.50 (0.20–30.53)	1.76 (0.13–23.32)	0.41 (0.03–6.61)	0.20 (0.01–4.01)	< 0.01	
Total energy intake levels (kcal)							0.25
n ≤ 1540	1(Ref)	0.66 (0.32–1.37)	0.84 (0.36–2.00)	0.87 (0.32–2.34)	0.21 (0.04–1.16)	0.28	
1540 < n ≤ 2206	1(Ref)	3.06 (1.24–7.51)	1.71 (0.63–4.66)	1.38 (0.43–4.44)	0.52 (0.10–2.63)	0.32	
n > 2206	1(Ref)	1.00 (0.34–2.91)	0.56 (0.19–1.64)	0.13 (0.04–0.48)	0.06 (0.01–0.28)	< 0.01	
Total protein intake levels (gm)							0.85
n ≤ 57.79	1(Ref)	0.80 (0.39–1.62)	1.21 (0.52–2.83)	0.87 (0.29–2.60)	0.39 (0.07–2.24)	0.62	
57.79 < n ≤ 87.36	1(Ref)	1.75 (0.76–4.05)	0.88 (0.35–2.22)	0.44 (0.15–1.29)	0.11 (0.02–0.55)	< 0.01	
n > 87.36	1(Ref)	0.98 (0.32–3.04)	0.67 (0.22–2.06)	0.38 (0.11–1.28)	0.13 (0.03–0.61)	< 0.01	
Total sugars intake levels (gm)							0.70
n ≤ 65.82	1(Ref)	0.96 (0.45–2.04)	1.07 (0.45–2.54)	1.21 (0.44–3.34)	0.11 (0.01–0.99)	0.39	
65.82 < n ≤ 115.17	1(Ref)	0.81 (0.35–1.86)	0.72 (0.29–1.80)	0.24 (0.08–0.72)	0.08 (0.02–0.39)	< 0.01	
n > 115.17	1(Ref)	2.47 (0.91–6.70)	0.98 (0.32–2.99)	0.37 (0.10–1.39)	0.29 (0.06–1.28)	< 0.01	
Total fat intake levels (gm)							0.47
n ≤ 52.93	1(Ref)	0.84 (0.42–1.69)	0.71 (0.30–1.67)	0.69 (0.25–1.88)	0.15 (0.03–0.85)	0.10	
52.93 < n ≤ 86.14	1(Ref)	1.08 (0.43–2.72)	1.32 (0.50–3.49)	1.00 (0.32–3.07)	0.26 (0.05–1.29)	0.25	
n > 86.14	1(Ref)	1.71 (0.61–4.79)	0.71 (0.23–2.14)	0.21 (0.06–0.76)	0.11 (0.02–0.53)	< 0.01	

Adjusted for age, gender, race, income, education, BMI, smoking status, drinking status, diabetes, hypertension, stroke, vigorous physical activity, total energy intake, total protein intake, total sugars intake, and total fat intake. “*P* for trend” is mainly used to test whether there is a certain linear change trend between the change in the exposure variable of HGS and the change in the outcome variable of HF among different subgroups. Interaction refers to the situation where the effect of one risk factor (A) on a certain disease outcome is different across strata of another risk factor (B), or vice versa. This means that if the interaction between A and B is present, A and B are not independent in causing a certain disease. “P for interaction” is mainly used to test whether the negative association between HGS and HF remains constant throughout all age groups. A *P* for interaction > 0.05 represents no interaction.

## Discussion

We comprehensively analyzed the covariates that may obstruct the identification of the association between HGS and HF, based on existing literature, clinical experience, and NHANES data. In this cross-sectional study, we revealed a negative association between HGS and HF in US adults older than 45, independent of age, gender, race, income, education, BMI, smoking status, drinking status, diabetes, hypertension, stroke, vigorous physical activity, total energy intake, total protein intake, total sugars intake, and total fat intake. No interactive role was found in the association between HGS and HF, suggesting that the above conclusions remained stable in the different subgroups.

Given the sensitivity to physiological changes, HGS was used as a valid marker of muscle function^36^. Although HGS has been found to be associated with mortality and the incidence of some cardiovascular diseases, the relationship between HGS and HF remains unclear^37^. Some studies have supported that HGS has no effect on the incidence of HF^15,16^. However, similar to our conclusion, a cohort study from England suggested that participants with a higher HGS had a lower incidence of HF. This conclusion was more obvious among participants aged > 65 years than among those aged ≤ 65 years in the subgroup analysis. However, the interaction between age and HGS for HF was not statistically significant^38^. The results of other studies based on the UK Biobank and Swedish National Inpatient Registry also revealed that objective measurements of HGS are strongly and independently associated with a lower HF incidence^39,40^. These conflicting conclusions may be attributed to the heterogeneity among these studies, including differences in participant selection, study size, study designs, and controlled covariates. Based on previous literature, our study excluded groups with depression and cognitive impairment, fully considered confounding factors, and strictly limited the age of the included population, making the conclusion reliable and filling in the gaps of current research from different perspectives.

Currently, no conclusive statement can be made about how muscle function decline could lead to the incidence of HF. From the existing literature, we speculate that the possible mechanism is as follows.

First, inflammation and oxidative stress might be an underlying mechanism for both muscle function decline and HF^41^. Inflammatory cytokines could alter blood vessel dynamics, which might result in alterations to muscle metabolism and muscle loss. For example, Wingless/Integrated (Wnt) signaling pathway molecules were found to play a critical role in tissue-specific stem cell aging and an increase in tissue fibrosis with age, involved in both calcification and loss of muscle mass, have been proposed as potential mediators^42,43^, In addition, the oxidative stress theory of aging suggested that age-associated functional losses were closely related to the accumulation of reactive oxygen and species (ROS)-induced damages^37^. Oxidative stress is involved in several age-related conditions including muscle function decline and HF^44,45^ The mechanism may be related to mitochondrial dysfunction leading to limited oxygen availability and subsequent reliance on anaerobic metabolism^46,47^.

Second, apoptosis may be another important underlying mechanism. Several apoptotic pathways have been linked with age-related muscle function^48^ and a higher frequency of myonuclear apoptosis has also been found in the muscle of patients with HF relative to age-matched healthy controls^49^.

Thirdly, abnormal glucose metabolism may be a common risk factor for HF and muscle function decline. Considering that skeletal muscle is the main site for insulin-mediated glucose disposal and that insulin resistance is strongly associated with HF, it could be hypothesized that insulin resistance plays a main role in both HF and muscle function decline^50^. In addition, it has been reported that muscular strength was demonstrated to be associated with a reduced risk of long-term development of diabetes mellitus^51^, which is known to be a major risk factor for the development of HF^52,53^.

Finally, muscle acted as a paracrine and exocrine organ, and the myokines may act in an autocrine, paracrine, and endocrine manner. The release of myokines from skeletal muscle preserves or augments cardiovascular function, in the meanwhile, increased muscle strength may provide capabilities for more active lifestyles that are related to a lower HF risk^40^.

To the best of our knowledge, our study is the first to provide evidence that HGS is negatively associated with HF among American middle-aged and elderly adults after properly identifying and adjusting covariates. The data collected in the NHANES is carried out following standardized protocols, and the NHANES is designed to provide nationally representative estimates. Therefore, the current findings have ideal generalizability. It is helpful for clinicians to identify groups at high risk for HF. To get higher-quality evidence, in the future, we intend to perform a cohort study utilizing local medical resources as well as a systematic review or meta-analysis to further investigate the association between HGS and HF.

There are also some limitations in our study. First, limited by the cross-sectional study design, this study had less power regarding the determination of causal relationships between HGS and HF. Second, since the study was conducted in a population of middle-aged and elderly individuals, the findings are only generalizable to relatively healthy adults. Third, although the NHANES considerably enhanced the reliability of the questionnaire survey by developing strict protocols, regular training of investigators, and other measures, recall bias, and self-report bias were still inescapable^54^. Fourth, while we controlled for a broad range of lifestyle and health-related factors, correcting for possible covariates remains challenging. Due to the limitations of the database, additional data on HF were not available to further stratify the patients. For example, ejection fraction, disease course, NT-proBNP, BNP, cardiac troponin, electrocardiogram, cardiac imaging data, and other diagnostic, and therapeutic indicators. In future studies, researchers should fully consider the above defects to provide higher-quality medical evidence.

## Conclusions

Overall, this cross-sectional study indicated that HGS was negatively associated with HF. This conclusion remained stable in participants aged ≥ 45 years, with different genders, races, incomes, education, BMI, smoking status, drinking status, diabetes status, hypertension status, stroke status, vigorous physical activity, total energy intake, total protein intake, total sugars intake, and total fat intake. This finding had the potential to draw attention to the physiological and pathological effects of decreased muscle function on HF and may influence further prospective studies with intervention trials. However, given the limitations of our study, this conclusion must be taken with caution.

## Data Availability

Data described in the manuscript, code book, and analytic code are available from the corresponding author on request.

## Abbreviations

HGS: Handgrip strength
HF: Heart failure
NHANES: The National Health and Nutrition Examination Survey
HFA: Heart Failure Association
ESC: European Society of Cardiology
NCHS: National Center for Health Statistics
STROBE: Strengthening the reporting of observational studies in epidemiology
BMI: Body mass index
BNP: B-type natriuretic peptide
NT-proBNP: N-terminal pro-B-type natriuretic peptide
SD: Standard deviation
IQR: Interquartile range
ORs: Odds ratios
CIs: Confidence intervals
ROS: Reactive oxygen and species
